# Gallstone Ileus Caused by Migration of Gallstone Through Cholecystoduodenal Fistula Resulting in Small Bowel Obstruction

**DOI:** 10.7759/cureus.37962

**Published:** 2023-04-21

**Authors:** Sukhjinder Chauhan, Rasiq Zackria, George Trad, Andrew Wojtanowski, John K Ryan

**Affiliations:** 1 Internal Medicine, Mountainview Hospital, Las Vegas, USA; 2 Gastroenterology, Sunrise Health GME Consortium, Las Vegas, USA; 3 Radiology, Mountainview Hospital, Las Vegas, USA; 4 Gastroenterology and Hepatology, Comprehensive Digestive Institute of Nevada, Las Vegas, USA

**Keywords:** cholelithiasis, enterolithotomy, small-bowel obstruction, choledochoduodenal fistula, gallstone ileus

## Abstract

Gallstone ileus is a rare condition characterized by mechanical obstruction of the intestine due to gallstone impaction. Diagnosis is based on clinical history, symptoms, and characteristic Computed Tomography (CT) scan findings. Treatment typically involves surgical extraction of gallstones, with laparoscopy as an effective and potentially safer approach. Here, we describe a case of an 84-year-old woman with gallstone ileus presenting with small bowel obstruction.

## Introduction

Gallstone ileus is a rare complication of cholelithiasis, in which a gallstone becomes lodged in the intestines causing an obstruction. It is observed in only 1%-4% of bowel obstruction cases and is common in older females [1]. The symptoms can be nonspecific and intermittent, leading to delayed diagnosis and high morbidity and mortality rates. Therefore, it is crucial to maintain a high level of suspicion for prompt identification and treatment of gallstone ileus [2, 3, 4]. In this case report, we describe an elderly female who developed small bowel obstruction due to a gallstone that migrated from the gallbladder through a cholecystoduodenal fistula.

## Case presentation

An 84-year-old woman presented to our hospital with a week-long history of abdominal pain, nausea, vomiting, and constipation. She was unable to tolerate oral intake due to worsening symptoms. She had no prior history of biliary colic or cholecystectomy but had a hiatal hernia noted on esophagogastroduodenoscopy (EGD) six months ago for gastroesophageal reflux disease (GERD). Her medical history included coronary artery disease, hypothyroidism, and an abdominal aortic aneurysm status post-endovascular repair. On admission, vital signs were within a normal range, but the physical examination was remarkable for epigastric tenderness and decreased bowel sounds. There was no rebound tenderness or guarding. Laboratory workup was unremarkable except for an elevated creatinine to 1.80 mg/dL, higher than her usual baseline of 0.80 mg/dL, consistent with acute kidney injury (AKI).

**Figure 1:**
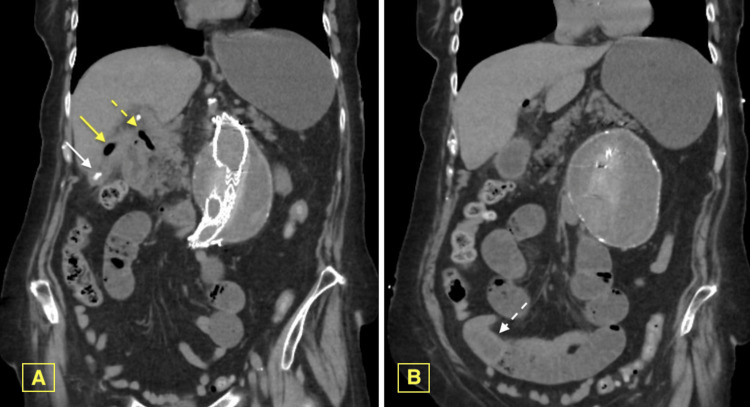
CT of abdomen and pelvis without IV contrast on admission. CT Abdomen Pelvis Non-Enhanced Imaging, A) Gallbladder intraluminal cholelithiasis (white arrow) and gas (yellow arrow). Extrahepatic bile duct pneumobilia (yellow dashed arrow). Inflammatory changes surrounding the gallbladder, involving the adjacent D1/D2 segments. B) Right lower quadrant small bowel transition point with rounded isodense mass (white dashed arrow) with proximal small bowel dilation. Note: both images demonstrate an abdominal aortic aneurysm with endovascular stenting.

A computed tomography (CT) abdominal and pelvis without contrast demonstrated intraluminal cholelithiasis and gas suggestive of pneumobilia with extrahepatic duct pneumobilia (Figure 1A). The surrounding area of the gallbladder was inflamed, and a rounded iso-dense mass in the right lower quadrant indicated a small bowel transition point with proximal small bowel dilation (Figure 1B).

**Figure 2:**
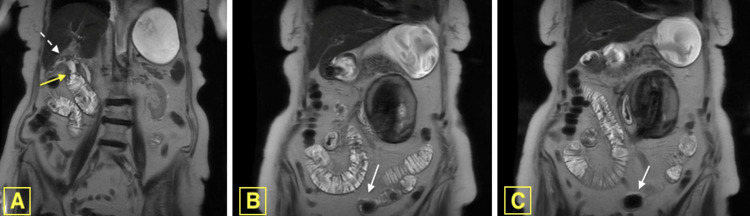
Magnetic resonance imaging (MRI) of the abdomen without contrast. Magnetic resonance imaging (MRI) of the abdomen without contrast, coronal single-shot fast spin echo (SS-FSE) sequence imaging. A) Cholecystoduodenal Fistula (yellow arrow) with several sub-centimeter calculi within the gall bladder (white dashed arrow). B-C) Multiple loops of small bowel dilation with a T2 hypointense rounded intraluminal mass (white arrow) at the small bowel transition point. Note: Both images demonstrate gastric and small bowel dilation.

Magnetic resonance imaging (MRI) of the abdomen without contrast demonstrated a cholecystoduodenal fistula and sub-centimeter calculi within the gallbladder (Figure 2A). Small bowel dilation and a T2 hypodense rounded intraluminal mass at the small bowel transition point were also observed (Figure 2B, 2C).

The patient subsequently underwent laparoscopic removal of the gallstone in the mid-ileus, cholecystectomy, and surgical repair of the duodenotomy fistula site. The surgery was well-tolerated by the patient. Samples from the cholecystectomy were evaluated for malignancy in the pathology lab. Immunohistochemical stains with Ki-67 and p53 showed no dysplasia, ruling out cholangiocarcinoma entirely.

## Discussion

Gallstone ileus, a rare condition complicating 0.3%-0.5% of cases of cholelithiasis with a female-to-male ratio of 3.5 to 1, was first described by Bartholin in 1654 during an autopsy [3, 5]. Gallstone ileus is a mechanical obstruction caused by a gallstone entering the GI tract through a fistula, often due to acute or chronic cholecystitis. Inflammation causes adhesions to adjacent tissue, which can result in pressure necrosis, erosion, and fistula formation. Gallstones may also pass into the duodenum through the common bile duct [6, 7]. Gallstones commonly lodge in the ileum (60.5% of cases) due to its narrow lumen and less active peristalsis. They can also impact the jejunum (16.1%), stomach (14.2%), colon (4.1%), and duodenum (3.5%). Gallstones larger than 2 cm are more likely to cause obstruction [7, 8]. The Bouveret Syndrome is a rare form of gallstone ileus, affecting 1-3.5% of cases, causing obstruction in the duodenum due to the blockage by a large gallstone in a wider part of the gastrointestinal tract [8, 9].

Gallstone ileus presents with symptoms often consistent with small bowel obstruction, including nausea, vomiting, constipation, dehydration, colicky abdominal pain, and abdominal distension. Symptoms can be intermittent due to the "tumbling phenomenon," where gallstones dislodge and travel distally, causing temporary relief of symptoms. Laboratory investigations may be unremarkable or nonspecific [4, 7]. A history of cholelithiasis should raise suspicion for gallstone ileus, but previous biliary symptoms are not always present in up to 50% of cases [9]. Plain abdominal films are often used to diagnose gallstone ileus but only show Rigler's triad (small bowel obstruction, pneumobilia, and ectopic radiopaque gallstones) in 14%-53% of cases. CT scans are commonly used for diagnosis, with high sensitivity and specificity, and can show Rigler's triad in 77.8% of cases [9]. MRI is useful in visualizing the biliary tree but is less readily available and not used in acute settings [9, 10].

Gallstone ileus requires surgical removal of the gallstone to relieve bowel obstruction. The three common surgical approaches include enterolithotomy alone, enterolithotomy combined with cholecystectomy and fistula closure (one-step surgery), and enterolithotomy followed by delayed cholecystectomy with fistula closure, usually 4-6 weeks later (two-step surgery) [11, 12]. Enterolithotomy alone is often used because of its lower complication rates and shorter hospital stays [11, 13]. One-step surgery reduces the risk of recurrence and other complications but has higher mortality and morbidity rates. Two-step surgery may increase the risk of complications while awaiting cholecystectomy and fistula repair [11, 12]. Gallstone ileus is usually treated with laparotomy, but it has high morbidity and mortality rates. Laparoscopy has emerged as a feasible alternative, with lower morbidity and faster recovery [11, 14, 15, 16]. Therapeutic endoscopy is an option for patients with colonic gallstone ileus or Bouveret syndrome, but it is technically challenging and unsuccessful in up to 91% of cases. Surgery is typically required [17, 18, 19].

Upon admission, our patient exhibited symptoms indicative of small bowel obstruction consistent with Rigler's triad, accompanied by characteristic imaging findings, culminating in a diagnosis of gallstone ileus. The patient underwent laparoscopic surgery that was completed without any issues and was successful in one step. This case highlights the importance of timely diagnosis and the safety and effectiveness of laparoscopic management for gallstone ileus.

## Conclusions

Gallstone ileus is a rare form of intestinal obstruction that can present with typical or nonspecific symptoms. It is important to maintain a high level of suspicion for prompt diagnosis, which is typically done through abdominal CT. Management involves surgical removal of the gallstone, which can be done through laparotomy or laparoscopy.
